# The relationship between tooth loss and hypertension: a systematic review and meta-analysis

**DOI:** 10.1038/s41598-022-17363-0

**Published:** 2022-08-03

**Authors:** Akio Tada, Rumi Tano, Hiroko Miura

**Affiliations:** 1 Faculty of Health Science, Hyogo University, 2301 Shinzaike Hiraoka-cho, Kakogawa, Hyogo 675-0195 Japan; 2 Department of Health Promotion, National Institute of Public Health, 2-3-6, Minami, Wako, Saitama 351-0197 Japan; 3 Division of Disease Control and Epidemiology, School of Dentistry, Health Sciences University of Hokkaido, 1757 Kanazawa, Ishikari-Tobetsu, Hokkaido, 061-0293 Japan

**Keywords:** Epidemiology, Dental public health, Dental epidemiology, Health care

## Abstract

As tooth loss is the high end of periodontal problems and edentulous individuals are at higher risk of nutritional problems like obesity, understanding the association between tooth loss and hypertension is important for improving cardiovascular health. We searched for publications from the last two decades using three electronic databases (PubMed, Web of Science and Scopus) and conducted a systematic review and meta-analysis on the association between tooth loss and hypertension according to PRISMA-P guidelines. Quality assessments were performed using the Newcastle–Ottawa Scale and the GRADE approach. Twenty-four studies (20 cross-sectional, and 4 cohort) met the inclusion criteria for this review. Most cross-sectional studies showed that subjects with more tooth loss exhibited a greater proportion of hypertension and higher systolic blood pressure than those with less tooth loss. Meta-analyses revealed a statistically significant association between tooth loss and hypertension. The pooled odds ratios of hypertension for having tooth loss with no tooth loss and for edentulous with dentate were 2.22 (95% CI 2.00–2.45) and 4.94 (95% CI 4.04–6.05), respectively. In cohort studies, subjects with more tooth loss had a greater incidence of hypertension than those with less tooth loss during the follow-up period. The present systematic review and meta-analysis suggests that tooth loss is associated with an increased risk of hypertension and higher systolic blood pressure.

## Introduction

### Background

Hypertension is a medical condition in which blood pressure is chronically 140/90 mmHg or higher^1^. The worldwide prevalence of hypertension has increased two-fold from 1990 to 2019, to more than 30% for both men and women aged 30–79^2^. Although high blood pressure does not typically cause symptoms, long-term high blood pressure is a major risk factor for coronary artery disease, stroke, heart failure, atrial fibrillation, peripheral arterial disease, vision loss, chronic kidney disease, and dementia^3–6^.

Risk factors for hypertension include obesity^7^, excessive intake of salt^8,9^, heavy alcohol consumption^10,11^, insufficient physical activity^12,13^, psycho-social stress^14,15^ and smoking^16^.

Several studies in dentistry have reported that patients with periodontitis have higher blood pressure^17–19^. It is estimated that severe periodontal diseases are prevalent in around 14% of the global adult population, representing more than one billion cases over the world^20^. Possible mechanisms for the association between periodontitis and hypertension are as follows: (1) periodontitis may elicit vascular inflammation which leads to endothelial dysfunction^21^; (2) periodontopathogens may stimulate local and systematic host immune responses, resulting in development of atherosclerosis^22^ and activate endothelial cells^23^; (3) the production of ROS increases in response to periodontal inflammation; subsequently ROS enter the systemic circulation^24^.

Tooth loss is the endpoint of progression of periodontitis. Tooth loss results in impaired mastication, which, in turn, makes it difficult to chew hard foods, consequently leads to deteriorated dietary habits^25^. Decline in masticatory function brings insufficient intake of vegetables and fruit and increased intake of fatty foods, which could cause obesity. The association between impaired mastication including tooth loss and obesity was suggested in a systematic review^26^. The relationship between obesity and hypertension has been demonstrated. It was reported that about half of new hypertension case attributed to overweight or obesity^27^. A meta-analysis showed that loss of body weight reduced blood pressure^28^_._ Although the impact of tooth loss on hypertension is estimated to be significant, no study has systematically reviewed articles that analyzed this association.

### Objective

The aim of this study was to systematically review the relationship between tooth loss/number of teeth and hypertension.

## Material and methods

This systematic review was structured following the PRISMA checklist. A protocol to address the a priori research questions, comprehensive literature search with inclusion and exclusion criteria for studies, screening methods, data abstraction, scientific study quality, and data analysis were developed to minimize bias.

### Literature search

The PICO model^29^ was used to select eligible studies in the present systematic review. The inclusion criteria were defined according to the population (P; “human adults”), intervention or exposure (I; “impact of tooth loss on hypertension”), comparison (C; “different number of remaining teeth, different number of missing teeth or dentate and edentulous”), and outcome (O; “hypertension”). The eligibility of the studies was assessed by three independent authors (Akio Tada, Rumi Tano and Hiroko Miura) through screening of titles and abstracts, according to the PICO model. The following PICO question was used: “Does tooth loss/number of teeth associate with hypertension?”. The inclusion criteria were defined as followed: (1) written in English, (2) published between 2001 and 2021, (3) investigating the association between tooth loss/number of teeth and hypertension, (4) conducted on adult subjects (age ≥ 18 years), and (5) using quantitative methods of data collection, were included in this review. The exclusion criteria were defined as followed: (1) subjects received oral and maxillofacial surgery or radiotherapy, (2) descriptive studies, reviews or studies with no analyses investigating the association between tooth loss and hypertension.

A literature search of the PubMed, Web of Science and Scopus databases was performed with relevant keywords (Mesh and non-Mesh). The search terms used were (“tooth loss” OR “number of teeth”) AND (“hypertension” OR “blood pressure”).

### Assessment of risk of bias in the included studies

For each selected observational study, the risk of bias was evaluated according to the criteria described by the Newcastle–Ottawa Scale (NOS) for cohort studies. This scale encompasses three domains: selection (four items), comparability (one item), and outcome (three items)^30^. Cross-sectional studies were graded as follows: very good, 9–10; good, 7–8; satisfactory, 5–6; unsatisfactory, 0–4^31^. Cohort studies were graded as follows: very good, 8–9; good, 7; satisfactory, 5–6; unsatisfactory, 0–4^32^.

The overall quality of evidence was evaluated using the Grading of Recommendation, Assessment, Development and Evaluation (GRADE) framework^33^. In this review, a narrative GRADE was chosen according to the types of studies included. Certainty of the evidence was evaluated in terms of study design, risk of bias, inconsistency, indirectness, and imprecision parameters, with categorization into one of four ratings: high, moderate, low, and very low^34^. Evidence issued in this review includes observational data, it started at low quality and then other issues within the magnitude of the effect, inconsistency, indirectness, imprecision, and counteracting plausible residual bias or confounding could be used to downgrade the evidence^35^. However, the quality of evidence can increase if studies strictly meet one of the following criteria: the magnitude of the treatment effect is very large. There is evidence of a dose–response relationship, or all plausible biases would decrease the magnitude of the treatment effect^36^.

### Data extraction

Data were extracted from each eligible study by three independent authors (Akio Tada, Rumi Tano, and Hiroko Miura) using a specifically developed data extraction sheet. Disagreements were resolved by consensus. The following data were extracted from each eligible study: first author, publication year, setting, type of study, number of subjects, confounding factors (demographic factors, socio-economic factors, health habits including smoking and alcohol consumption, and systemic disease such as diabetes, hypercholesterolemia, and obesity), and outcome measures including both adjusted odds ratios and 95% confidence intervals (CIs).

### Statistical analysis

Meta-analyses were conducted using a random-effects model. Studies were excluded if they did not report an outcome in each group or did not have enough information available to calculate the OR. The numbers of subjects according to the status of the remaining teeth and the presence of hypertension were extracted from each study. A separate meta-analysis was performed for each outcome variable. Effect sizes were reported as pooled ORs with 95% CIs for categorical outcomes. The heterogeneity of the effect size estimates across these studies was quantified using the I^2^ statistic. The I^2^ statistic ranges from 0 to 100% (I^2^ < 25%, low heterogeneity; I^2^ = 25–50%, moderate heterogeneity; and I^2^ ≥ 50%, substantial heterogeneity)^37^. All data analyses were performed using STATA version 16.

## Results

### Literature searches and study characteristics

**Figure 1:**
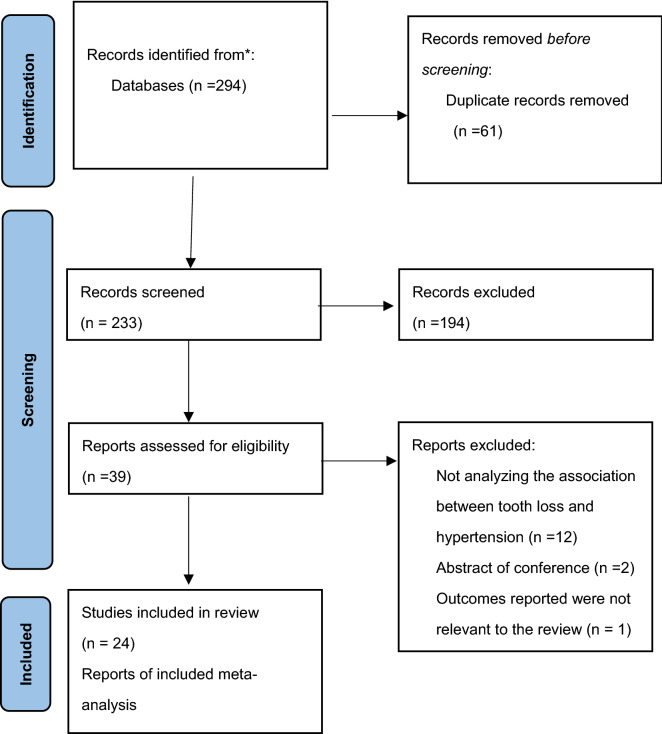
Flow diagram of literature search.

The initial comprehensive literature search identified a total of 294 articles; eligible articles were retrieved through a manual search (Fig. 1). After the removal of duplicates, the titles and abstracts of 233 records were screened. Of these, 194 articles were excluded according to the exclusion criteria written in the “Material and methods”. The remaining 39 articles were screened for further analyses as follows.

Of the 39 potentially relevant articles, 15 were excluded because they did not meet the inclusion criteria. Most of excluded studies did not analyze the association between tooth loss and hypertension. Finally, a total of 24 publications (20 cross-sectional studies^38–57^, and four cohort studies^58–60^) were included in this systematic review, as shown in the flow chart (Fig. 1).

Characteristics of studies are presented in Table 1. Eighteen studies^38,41–46,48–50,52,54,56–60^ categorized blood pressure into hypertensive and normal according to global criteria^62^ while six studies^39,40,47,51,53,55^ used values of systolic blood pressure (SBP) and diastolic blood pressure (DBP) and did not apply these categories. Four studies used self-reported information on hypertensive status^42,50,54,60^.

**Table 1:** Summary of studies on the relationship between tooth loss/number of teeth and hypertension.

**(1) Cross-sectional study for the association between tooth loss/number of teeth and hypertension**						
References	Study sample	Tooth loss/number of teeth	Hypertensive status	Control of confounding factors^a^	Key results	
Mendes et al.^38^	10,576 patients from university clinic (dental) aged 18 years and older (Portugal)	No. of missing teeth	Hypertension Category: Hypertension/Normotensive Systolic blood pressure (SBP) Diastolic blood pressure (DBP) Mean ± SD	1, 3, 4	One tooth loss increased hypertension (adjusted OR (aOR) 1.04; 95% CI 1.03–1.04)	
Hosadurga et al.^39^	270 outpatients aged 20–59 (Malaysia)	No. of missing teeth Category: edentulous/partial tooth loss	SBP DBP Mean ± SD	1, 2, 3, 4	In multivariable linear regression models, there was no significant association between tooth loss and SBP and DBP	
Del Brutto et al.^40^	1543 community dwelling aged 40 years and older (Ecuador) ≥	No. of remaining teeth Category: 10 ≥ /10 <	SBP DBP	1, 2, 3, 4	Significance association between edenturism and hypertension was not observed in adjusted generalized linear models	
Da et al.^41^	3677 community dwelling aged 50 years and older (China)	No. of missing teeth Category: ≤ 3/4–14/ ≥ 15	Hypertension Category: hypertension/normotensive	1, 2, 3, 4	Individuals with ≥ 15 missing teeth have significantly higher risk of stage III hyper tension than those with ≤ 3 (aOR 1.03; 95% CI 1.03–1.64)	
Dar-Odeh et al.^42^	10,576 female patients from university clinic (dental) aged 18 years and older (Saudi Arabia)	No. of missing teeth Category:No missing teeth/Having missing teeth	Hypertension Category: hypertension/normotensive	1, 3, 4	In a linear regression model, missing teeth was marginally significantly associated with hypertension (p = 0.088)	
Al-Ahmad et al.^43^	60 postmenopausal women (Malaysia)	No. of missing teeth	Hypertension Category: hypertension/normotensive		Postmenopausal women with hypertension showed more significant tooth loss compared to those with normal tension (p < 0.05)	
Delgado-Perez et al.^44^	60 patients in a health center (Mexico)	No. of missing teeth Category: no missing teeth/Having missing teeth	Hypertension Category: hypertension/normotensive	1, 2,	Individuals with hypertension had higher risk of more missing teeth (incidence rate ratios [IRRs] = 2.63;95% CI 1.77–3.90)	
Gordon et al.^45^	1341 postmenopausal women (US)	No. of missing teeth	Hypertension Category: hypertension/normotensive SBP, DBP	1, 2, 3,4	In a linear regression model, number of missing teeth was significantly associated with hypertension (p = 0.01)	
Shin^46^	13,651 commuity dwelling aged 19 years and older Data from the 2015 Korean National Health and Nutrition Examination Survey (South Korea)	No. of remaining teeth Category:0/1–19/20–27 /28	SBP, DBP Hypertension Category: hypertension/normotensive	1, 2, 3, 4	Subjects with tooth loss have significantly higher risk of hypertension than those with 28 teeth (0 aOR 1.63; 95% CI 1.22–2.18, 1–19 aOR 1.46; 95% CI 1.22–1.76; 20–27 aOR 1.25; 95% CI 1.11–1.43)	
Moghadam et al.^47^	700 community dwellings aged 35 years and older (Iran)	No. of missing teeth	SBP, DBP	1, 2, 3, 4	In multivariable linear regression models, number of missing teeth was significantly associated with SBP (p = 0.01) and DBP (p = 0.03)	
Laguzzi et al.^48^	341 community dwellings aged 15–24, 35–44, 65–74 years (Urugay)	No. of remaining teeth Category: having 20 teeth/ edentulism	Hypertension Category: hypertension/normotensive	1, 2, 3, 4	No association between tooth loss and hypertension	
Kim et al.^49^	8058 community dwellings aged 40 years and older Data from the 2012 Korean National Health and Nutrition Examination Survey (South Korea)	No. of remaining teeth Category:0–19/20–27/28	Hypertension Category: hypertension/normotensive	1, 2, 3, 4	Women with 0–19 and 20–27 teeth have significantly higher risk of hypertension than those with 28 teeth (0–19 aOR 1.57; 95% CI 1.07–2.31, 20–27 aOR 1.41; 95% CI 1.08–1.84). No significant difference was found in men	
Singh et al ^50^	1486 community dwellings aged 45 years and older (India)	No. of missing teeth Category: Having no tooth loss/Having some tooth loss/Edentulous	Hypertension Category: hypertension/normotensive	1, 2, 3, 4	Individuals with tooth loss had higher risk of hypertension than those with no tooth loss (aOR 1.62: 95% CI 1.12–2.35)	
Darnaud et al.^51^	102,330 individuals, who underwent medical and oral examinations (France)	No. of missing teeth Category: 10 ≥ /10 <	SBP, DBP	1, 3, 4	Individuals < 65 years with missing teeth > 10 had significantly higher risk of high blood pressure (≥ 140 mmHg) than counterpart (aOR = 1.17; 95% CI 1.07–1.31)	
Zhu et al^52^	5511 community dwellings aged (US)	No. of remaining teeth Category:0/1–20/21–27/28	Hypertension Category: hypertension/normotensive SBP, DBP	1, 2, 3	Edentulous subjects have significantly higher risk of hypertension than those with 28 teeth (aOR 1.45; 95% CI 1.13–1.87)	
Peres et al.^53^	1720 community dwelling aged 20–59 years (Brazil)	No. of remaining teeth Category: < 10 teeth at least in one arch/ ≥ 10 in both arches/ Edentulous	SBP, DBP	1, 2, 3, 4	Edentulous subject had a SBP 8.3 mmHg (95% CI 0.1; 16.7) higher than those with more than 10 teeth in both arches after adjustment for potential confounders	
Islas-Granillo et al.^54^	139 elderly who resided at long term facility or attended adult day center aged 60 years and older (Mexico)	No. of remaining teeth Category: Having no teeth/Having teeth	Hypertension Category: hypertension/normotensive	1	Being edentate has a higher risk of hypertension with approaching significance (p = 0.067)	
Lee et al.^55^	3611 community dwelling aged 60 years and over (Korea)	No. of missing teeth Category: < 8/9–19/19–28	SBP DBP	1, 2, 3, 4	In the linear logistic regression model, SBP was positively significantly associated with number of missing teeth (p < 0.001)	
Völzke et al.^56^	4185 community dwelling aged 20–79 years (Germany)	No. of remaining teeth Category: 0–6/7–18/19–23/24–26/27–28	Hypertension Category: hypertension/normotensive SBP DBP	1, 2, 3, 4	Men with 0–6 and 7–18 teeth have significantly higher risk of hypertension than those with 27–28 teeth (0–6 aOR 1.91; 95% CI 1.21–3.02, 7–18 aOR 1.81; 95% CI 1.08–2.39). No significant difference was found in women	
Taguchi et al.^57^	67 postmenopausal women (Japan)	No. of missing teeth Category: Having missing teeth/no missing teeth	Hypertension SBP DBP	4	Subjects with missing teeth have significantly higher risk of hypertension than those with no missing. (aOR = 3.59; 95% CI 1.10–11.7)	

**(2) Cohort study for the association between loss/number of teeth and hypertension**						
References	Study sample	Study period	Tooth loss/number of teeth	Hypertension status	Control of confounding factors^a^	Key results
Woo et al.^58^	19,680 community dwellings with a mean age of 51.8 (South Korean)	7 years	No. of missing teeth Category: 0/1–7/8–14/ ≥ 15	Hypertension Category: hypertension/normotensive	1, 2, 3, 4,	Subjects with ≥ missing teeth have significantly higher risk of incidence of hypertension than those with no missing teeth (adjusted hazard ratio (aHR) 2.26; 95% CI 1.24–4.10)
Gordon et al.^61^	36,692 postmenopausal women (US)	8.3 years	No. of remaining teeth Category: having no teeth/having teeth	Hypertension Category: hypertension/normotensive	1, 2,3, 4	Edentuous subjects have significantly higher risk of incidence of hypertension than dentate (aHR 1.21; 95% CI); 1.211.11–1.30)
Kim et al.^59^	514,866 community dwellings aged 40–79 (South Korean)	10 years	No. of tooth loss during study period	Hypertension Category: hypertension/normotensive	1, 2, 3, 4,	Subjects with hypertension showed 0.97–0.94-fold increased risk of experiencing a loss of ≥ 4 and 2–3 teeth
Rivas-Tumanyan et al.^60^	31,543 male health professionals aged 40–79 (US)	20 years	No. of remaining teeth Category: 0–10/11–16/17–24/25–32	Hypertension Category: hypertension/normotensive	1, 2, 3, 4	Significant associations between incident hypertension and tooth loss during follow-up (RR = 1.03; 95% CI: 0.98–1.09) was not observed

^a^The following variables were controlled for in the analyses or with separate results: 1, demographic factors; 2, socio-economic factors; 3, smoking/alcohol; 4, diabetes, hypercholesterolemia, and obesity.

In terms of the number of teeth, 14 studies evaluated the number of missing teeth^38,39,41–45,47,50,51,55,57,58,59^ while 10 studies evaluated the number of remaining teeth^40,46,48,49,52–54,56,61,60^_._ In the analyses, only four studies used measured values^43–45,47^ while the other 20 categorized measured values. Categorizations were vastly different.

Regarding the age range of subjects, 8 studies included patients < 40 years^38,39,42,46–48,53,56^. Four studies used menopausal women^43,45,57,61^.

The distribution of countries where the included studies were conducted is as follows: Asia, 12; Central-South America, 5; North America, 4; and Europe 3.

### Quality evaluation for each article

The quality of the studies was evaluated using NOS scores (Table 2). Seven studies were classified as “very good”^40,45,46,48,55,56,58^, 12 as “good”^38,39,41,47–51,53,57,61,59^, four as “satisfactory”^42–44,60^ and one as “unsatisfactory”^54^.

**Table 2:** Newcastle–Ottawa Scale score of included studies.

**(1) Cross-sectional study**										
References	Selection				Comparability	Outcome		Score	Evaluation	
	1	2	3	4		1	2			
Mendes et al.^38^		*		**	*	**	*	7	Good	
Hosadurga et al. ^39^	*	*		*	**	**	*	8	Good	
Del Brutto et al. ^40^	*	*		**	**	**	*	9	Very good	
Da et al.^41^	*	*		*	**	**	*	7	Good	
Dar-Odeh et al.^42^		*		**	*	*	*	6	Satisfactory	
Al-Ahmad et al.^43^	*			**		**	*	6	Satisfactory	
Delgado-Perez et al. ^44^				**	*	*:	*	5	Satisfactory	
Gordon et al.^45^	*	*		**	**	**	*	9	Very good	
Shin ^46^	*	*		**	**	**	*	9	Very good	
Moghadam et al. ^47^	*	*		*	**	*:	*	7	Good	
Laguzzi et al.^48^	*	*		**	**	**	*	9	Very good	
Kim et al.^49^	*	*		**	**	**	*	8	Good	
Singh et al.^50^	*	*		*	**	*	*	7	Good	
Darnaud et al. ^51^		*		**	*	**	*	7	Good	
Zhu et al. ^52^	*	*		**	*	**	*	8	Good	
Peres et al.^53^	*	*		*	**	**	*	8	Good	
Islas-Granillo et al.^54^				**		*	*	4	Unsatisfactory	
Lee et al.^55^	*	*		**	**	**	*	9	Very good	
Völzke et al. ^56^	*	*		**	**	**	*	9	Very Good	
Taguchi et al.^57^	*			**	*	**	*	7	good	

**(2) Cohort study**										
References	Selection				Comparability	Outcome			Score	Evaluation
	1	2	3	4		1	2	3		
Woo et al.^58^	*	*	*	**	**	*	*		9	Very good
Gordon et al.^61^	*	*		*	**	*	*		7	Good
Kim et al.^59^	*	*	*		**	*	*		8	Good
Rivas-Tumanyan et al.^60^		*		*	**		*	*	6	Satisfactory

### Quality evaluation for evidence

The overall certainty of the two pieces of evidence was evaluated using the GRADE system. The certainty of evidences stayed in “Low”, initial rating of observational studies, with no upgrading and downgrading shown as shown in Table 3.

**Table 3:** Summary of evidences according to GRADE approach.

No of studies	Study design	Risk of bias	Inconsistency	Indirectness	Imprecision	Other consideration	Certainty
**Association between tooth loss and hypertension**							
16	Observational studies	Not serious	Not serious	Not serious	Not serious		Low
**Association between tooth loss and blood pressure**							
6	Observational studies	Not serious	Not serious	Not serious	Not serious		Low

### Association between number of remaining/missing teeth and hypertension

The prevalence of hypertension was compared between/among groups with different number of remaining teeth in six cross-sectional studies^46,48,49,52,54,56^ (Table 1). Four of the six studies showed negatively significant associations between the number of remaining teeth and hypertension after controlling for confounders^46,49,52,56^. Another study found that the association was slightly below the threshold of statistical significance^54^. On the other hand, a different study failed to find an association between the number of remaining teeth and hypertension^48^.

Six cross-sectional studies analyzed the association between number of missing teeth and hypertension^38,41–44,50^ (Table 1). Two studies showed that groups with hypertension had higher mean^43^ or median^44^ of number of missing teeth than normal blood pressure group. Four of the six studies displayed that positively significant associations after adjusting with confounding factors^38,41,45,50^. One study showed that menopausal women with hypertension had a higher number of remaining teeth than those without hypertension^43^. In Dar-Odeh’s study, the association was marginally significant^42^.

### Association between number of remaining/missing teeth and SBP/DBP

Four studies analyzed the associations between number of remaining teeth and SBP/DBP^40,52,53,56^ (Table 1). Two studies demonstrated that the number of remaining teeth was inversely associated with SBP and DBP even after adjusting for covariates^52,53^. In Holzke’s study, men with fewer teeth had significantly higher SBP than those with higher teeth was significantly higher, but this association was not observed in women^56^. Another study showed that the population with a higher number of remaining teeth exhibited a significantly higher SBP than the population with a lower number of remaining teeth, but this association was not observed with DBP^40^. However, the significance disappeared after adjusting for covariates.

A further six studies analyzed the associations between number of missing teeth and SBP/DBP^39,45,47,51,55,57^ (Table 1). Two studies reported that populations with a greater number of missing teeth exhibited significantly higher SBP or DBP than those with a smaller number of missing teeth^39,51^ but this significance disappeared after adjustment for potential confounding factors. On the other hand, two studies found that a population with more missing teeth had significantly higher SBP and DBP than the population with fewer missing teeth after adjusting the confounding factors^47,55^. In Darnard’s study^51^, a sub-population with missing teeth > 10 was 1.17 times more likely to have SBP > 140 mmHg than the sub-population with missing teeth ≤10 among adults aged < 65 years. However, this association was not observed among adults aged ≥ 65 years.

### Cohort study

Four studies performed cohort study^58–60^ (Table 1). Three studies have compared incidence of hypertension in regard to different number of teeth^58–60^. One study made a comparison between subjects who are edentulous and those who are dentate^61^. Edentulous participants had a significantly higher risk of incidence of hypertension after adjusting for confounders. Two study analyzed the association between the number of teeth at baseline and the incidence of hypertension during follow-up. A significant higher incidence of hypertension was observed in population with lower number of teeth in one study^58^ but not in another^60^ after adjusting for confounders. Kim et al. compared missing teeth between subjects who had hypertension and those who did not at baseline^59^. In univariate analysis, the hypertension group had a higher risk of tooth loss than the normal group. However, in the multivariable analysis, the opposite result was observed.

### Meta-analysis

**Figure 2:**
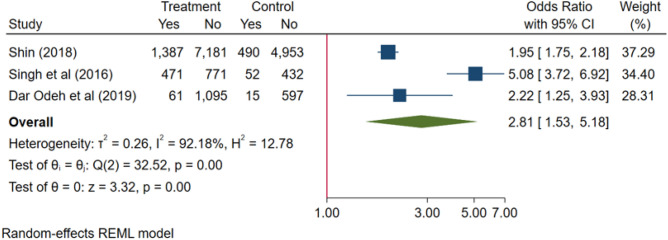
Forest plots of the Odds ratios with corresponding 95% Cis of studies on non-tooth loss/tooth loss and risk of hypertension.

**Figure 3:**
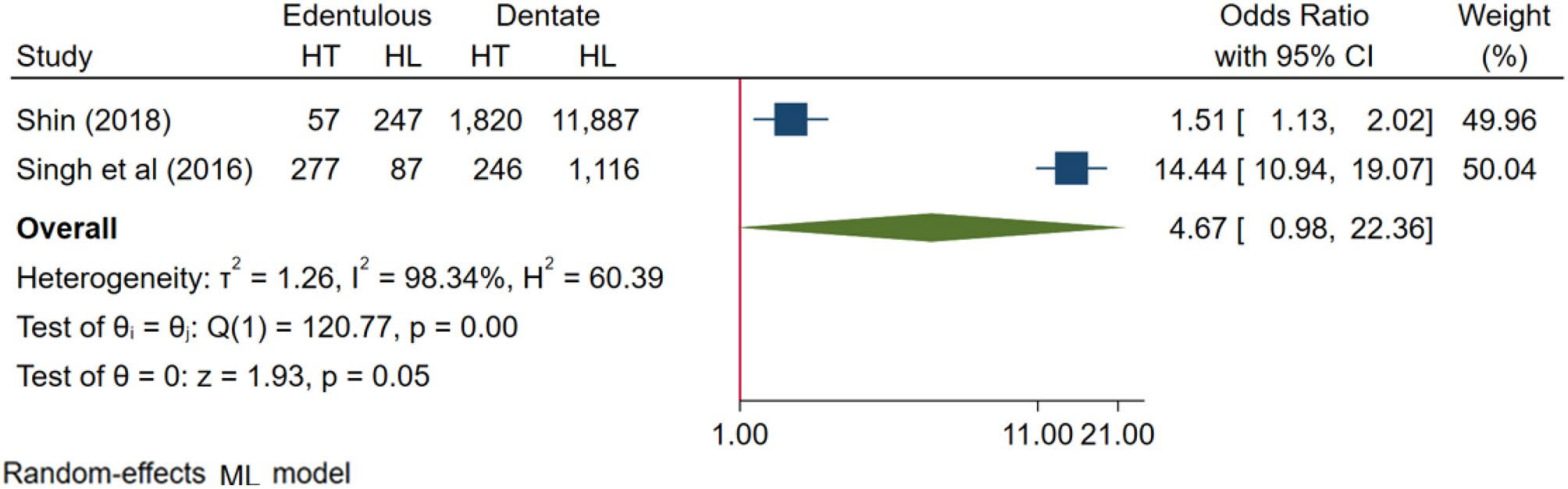
Forest plots of the Odds ratios with corresponding 95% Cis of studies on dentate/edentulous and risk of hypertension.

Separate meta-analyses regarding the risk of hypertension were carried out for “no tooth loss” versus “tooth loss” and for “dentate” versus “edentulous”. Three studies entered in the meta-analysis and found that those with tooth loss have a significantly higher prevalence of hypertension than those with no tooth loss^42,46,50^. Based on the data of the studies for “no tooth loss” versus “tooth loss”, the pooled summary odds ratio was 2.22 (95% CI 2.00–2.45) in the random-effect model for the group with tooth loss compared to the non-tooth loss group (Fig. 2). In other words, the tooth loss group was 2.22-fold more likely to be diagnosed with hypertension. As for “dentate” versus “edentulous”, two studies found that edentulous subjects had a significantly higher prevalence of hypertension than dentate subjects^46,50^ and the pooled summary OR was 4.94 (95% CI 4.04–6.05) in the random-effect model for the edentulous group compared to the dentate group (Fig. 3). These findings were statistically significant (*p* < 0.001). However, the statistical heterogeneity was high across all studies (*I*^2^ = 94.8% and 99.1%, respectively).

## Discussion

### Impact of tooth loss on hypertension

Most of the included studies have demonstrated an association between tooth loss and hypertension with individuals having greater tooth loss exhibiting a higher prevalence of hypertension and higher blood pressure. Only two studies reported no association between tooth loss and hypertension without adjusting for possible confounding factors. Evidence from our literature review suggested that tooth loss is thought to have a significant association with hypertension. However, a few studies reported that this significant association disappeared after adjusting with possible confounding factors and the odds ratios for this association in most studies, which ranges between 1 and 2, are not very large. Therefore, the extent of the association between tooth loss and hypertension may be varied.

There are two possible cascades related to the association between tooth loss and hypertension. It is speculated that progression of periodontitis, a major cause of tooth loss, consequently results in hypertension. The mechanisms by which periodontitis elicits hypertension are complex and not fully elucidated. The major mechanism by which increased blood pressure occurs in patients with periodontitis is likely to be the spread of inflammation and secondary damage to the vascular endothelium^63–66^. Periodontal tissue covers a wide area of the oral cavity. The influence of local inflammation of periodontitis occurring in a large proportion of the oral cavity may significantly contribute to systemic inflammation mediated by C-reactive protein and main inflammatory cytokines such as tumor necrosis factor alpha, interleukin 1b and interleukin 6^67,68^. Increases in nitrate-reducing bacteria, which are observed in patients with periodontitis may induce a reduction of nitric oxide, which may consequently lead to an increase in blood pressure^69–71^. It was also reported that an intervention of non-surgical periodontal treatment leads to an improvement of both periodontal status and hypertension^72^. Another reason is that tooth loss causes a decrease in masticatory function, thereby inducing obesity. There are two possible explanations for the association between mastication and obesity. One is that the eating habits of people with poor masticatory function, and decreased consumption of vegetables and fruits, and higher consumption of high energy food, tend to cause obesity compared to those with adequate mastication^73–76^. Another is that a reduction in chewing leads to a decrease in diet-induced thermogenesis and inactivation of neuronal histamine, which may consequently lead to obesity^77–79^.

In patients with metabolic syndrome, other factors, such as diabetes and hyperlipidemia, add to this complex relationship. Obesity is one of the causes of diabetes^80^ and diabetes and periodontitis have a bidirectional relationship^81^. In some of the studies reviewed in this article, the association between tooth loss and hypertension disappeared after adjustment for confounders (demographic factors, socio-economic factors, health behavior and general health). This suggests that other factors have a considerable influence on this association. It is thought that various factors interact with each other in a complicated cascade from tooth loss to hypertension.

This systematic review aimed to analyze the association between tooth loss and hypertension by systematically summarizing the scientific evidence from clinical studies during the last two decades. Twenty-four studies were included, with a total of 772,683 adults. To the best of our knowledge, this is the first systematic literature review to examine this association. The results of this review may help elucidate the influence of oral health on blood pressure.

### Comparison of the association between tooth loss and hypertension

The association between tooth loss and hypertension was compared for each geographical location. Studies conducted in Central-South America showed a trend for weaker association compared to other areas; one with no association^48^, one with approaching significance level (p = 0.067)^54^, and one with disappearance of significance after adjustment with confounders^40^ in 5 studies. The reason for the difference is unclear. In Central-South America studies, studies from this region showed higher tooth loss than those from other areas’ studies, particularly developed countries. Tooth loss in younger people may not cause hypertension easily. More detailed analysis is needed.

There was a difference in the association between tooth loss and hypertension between men and women. For women, the association between tooth loss and hypertension was marginally or not observed in studies including younger subjects (19–39 years)^42,56^. On the other hand, significant associations were found in studies with subjects aged 40 or older years after adjusting confounders^44,47,49,59^. It is thought that younger subjects with tooth loss may have smaller prevalence of hypertension than older subjects with tooth loss. However, in the Völzke’s study including individuals aged 20–79 years, the significant association was found among men unlike among women^56^. In a Korean study, men aged 40 and older did not show the significant association unlike women^49^. Various factors may influence this association.

### Difference in the association with hypertension between SBP and DBP

Although a significant association between SBP and tooth loss was observed, the association between DBP and tooth loss was not significant. The reasons why SBP exhibits a stronger association with tooth loss than DBP is unclear. The proportion of individuals with diastolic hypertension (systolic–diastolic hypertension [SDH] or isolated diastolic hypertension [IDH]) decreased, while that of those with systolic hypertension increased with age. Accumulation of advanced glycation end products (AGE) with aging leads to increased arterial stiffness and contributes to the development of isolated systolic hypertension^82,83^. Excessive intake of animal-derived foods which are rich in fat and AGE may increase the risk of hypertension and other chronic disease^84–87^. In general, since individuals who suffer from decreased masticatory function due to tooth loss tend to eat foods that are high in fat^88–91^, this may induce AGE accumulation, consequently contribution to increased SBP.

### Cohort studies

Two prospective cohort studies demonstrated an increased incidence of hypertension in individuals with more tooth loss during the observation period^58,61^. On the contrary, one study reported that subjects with hypertension experienced lower tooth loss than those without hypertension^59^. Therefore, the association between tooth loss and hypertension may not be bidirectional. In other words, hypertension may not cause tooth loss.

### Quality of studies

Of the 24 included studies, 16 (66.7%) adjusted the association between blood pressure and tooth loss with all possible confounding factors^39–41,45–50,53,55,56,58–60^. Of the remaining eight studies, 4 studies lacked adjustment for only one confounding facto^38,42,51,52^. The most important confounding factor was obesity, and almost all studies adjusted for this. These adjustments strengthen the reliability of the evidence obtained in this review. However, three studies that investigated patients from university hospitals failed to adjust for socio-economic factors^38,42,51^. The characteristics of these studies’ settings might make it difficult to obtain data of socio-economic factors.

Eight studies employed self-reported data on the number of teeth and/or hypertension. Some studies have shown strong validity of self-reported number of tooth loss in high-income countries^88,89^. One study examined in health professionals in the US, expecting high validity against clinically measured results^60^. However, the validity of the self-reported number of lost teeth has not been evaluated in lower and middle-income countries. Moreover, the validity of self-reported hypertension in developing countries is not high^90,91^. Since most of the studies that employed self-reported data on the number of teeth and/or hypertension in this review were performed in developing countries, self-reported data may deviate from true values. Since subjective measurements have the possibility to give considerable optimistic results compared with practitioners’ measurements^92^, self-reported data may often be underestimated.

### Strengths and limitations

There are several limitations in this study. First, all studies included in this review were observational studies. Intervention studies are necessary to analyze the causal relationships.

Second, the grouping of participants according to the number of teeth differed among the studies. Due to this problem, only small-scale meta-analyses were performed. Moreover, the only meta-analyses that were carried out were to compare the hypertension rate for tooth loss vs. no tooth loss and for dentate vs. edentulous. Pooled odds ratio data based on a cut-off value for the number of teeth is valuable for estimating the association between the number of remaining teeth and hypertension. Moreover, it may be a rough indication for maintaining oral health to prevent hypertension.

Third, some studies could not be included in meta-analysis because of lack of data for real number of subjects by tooth loss and hypertension. This lack lowers the value of results of meta-analysis. Nonetheless, odds ratios for the association between tooth loss and hypertension of these studies are similar to those of studies included meta-analysis. It is thought that missing of these data would give little influence on pooled ORs.

Forth, several studies have investigated many specialized subjects, including patients from clinics or hospitals^38,39,42,44,54^, menopausal women^43,45,57,61^, and male health care specialists^60^. Although it is problematic to apply the results of these studies to the general population, the large number of subjects in their studies increases the reliability of the study results.

The strength of our study is that it included many studies with a large number of subjects. Eleven of the 24 included studies have investigated large-scale community dwellings of > 1000 individuals^40,41,46,49,50,52,53,55,56,58,59^, which enhances the credibility of the results of the studies. The greater the sample size, the smaller the error, which makes the results more reliable.

### Future direction

Although lost teeth cannot be regenerated, they can be restored by prosthetics. Provision of prosthetics can improve both masticatory function and diet. When decreased mastication elicits obesity and subsequently hypertension, the provision of prosthetics may improve increased blood hypertension. The number of teeth acts only as an anatomical indicator. There is an indicator, functional teeth that is the sum of the number of natural teeth and the number of lost teeth that are restored by prosthetics. It is of interest to investigate the association between the number of functional teeth and hypertension.

The lack of standardized division criteria for tooth loss makes it impossible to compare the strength of associations between tooth loss and hypertension observed in reviewed studies. The unification of division standards for tooth loss should be proposed.

## Conclusion

In the present review, we provided an overview and appraisal of studies regarding the relationship between the number of teeth/tooth loss and hypertension. People with fewer remaining teeth or more tooth loss exhibited a higher prevalence of hypertension. Those with more tooth loss had significantly higher SBP but not DBP than those with less tooth loss. Those with more tooth loss showed a higher incidence of hypertension than those with less tooth loss during the observation period (Supplementary Information).

## Supplementary Information

Supplementary Information 1.

Supplementary Information 2.

Supplementary Information 3.

## Data Availability

The datasets used and/or analysed during the current study available from the corresponding author on reasonable request.
