# Supplementary material for: The relationship between tooth loss and hypertension: a systematic review and meta-analysis
Source: Sci Rep. 2022 Aug 3;12:13311. doi: 10.1038/s41598-022-17363-0 (PMC9349209; doi:10.1038/s41598-022-17363-0)
Supplement: Supplementary file 1 — Supplementary Information 1. [file 41598_2022_17363_MOESM1_ESM.docx]

List of Digital Object Identifier (doi) of references

| References | doi |
| --- | --- |
| Mendes *et al*.^37^ | 10.3390/ijerph18010285. |
| Hosadurga *et al.* ^38^ | 10.4103/jfmpc.jfmpc_811_19. |
| Del Brutto *et al.* ^39^ | 10.1177/2150132720928670. |
| Da *et al*.^40^ | 10.1186/s12903-019-0966-3. |
| Dar-Odeh *et al*.^41^ | 10.1177/2150132720928670. |
| Al-Ahmad *et al.*^42^ | 10.4103/ejd.ejd_322_17. |
| Delgado-Perez *et al.* ^43^ | 10.4103/njcp.njcp_52_17. |
| Gordon *et al.*^44^ | 10.1002/JPER.17-0562. |
| Shin ^45^ | 10.1002/JPER.17-0413. |
| Moghadam *et al*. ^46^ | https://www.rjpbcs.com/archives.html* |
| Laguzzi *et al.*^47^ | 10.1111/jphd.12123. |
| Kim *et al*.^48^ | [10.1097/MD.0000000000003331](https://dx.doi.org/10.1097/MD.0000000000003331) |
| Singh *et al* ^49^ | 10.1111/jphd.12136. |
| Darnaud *et al.* ^50^ | [10.1093/ajh/hpv025](https://doi.org/10.1093/ajh/hpv025) |
| Zhu *et al* ^51^ | 10.1111/jcpe.12361. |
| Peres *et al*.^52^ | 10.1111/j.1600-051X.2012.01916.x. |
| Islas-Granillo *et al.*^53^ | 10.1016/j.archger.2010.12.014. |
| Lee *et al* ^54^ | 10.1016/j.archger.2009.01.005. |
| Völzke *et al* ^55^ | 10.1097/01.hjh.0000234104.15992.df. |
| Taguchi *et al.*^56^ | 10.1161/01.HYP.0000128335.45571.ce. |
| Woo *et al.*^57^ | 10.1371/journal.pone.0253257. |
| Gordon *et al.*^58^ | 10.1093/ajh/hpy164. |
| Kim *et al.*^59^ | 10.5051/jpis.2019.49.3.158. |
| Rivas-Tumanyan *et al.*^60^ | 10.1038/ajh.2012.32. |

*:Since doi was not found, we indicated Hypertext Transfer Protocol Secure of the site that includes PDF file of the reference
