# Supplementary material for: The relationship between tooth loss and hypertension: a systematic review and meta-analysis
Source: Sci Rep. 2022 Aug 3;12:13311. doi: 10.1038/s41598-022-17363-0 (PMC9349209; doi:10.1038/s41598-022-17363-0)
Supplement: Supplementary file 3 — Supplementary Information 3. [file 41598_2022_17363_MOESM3_ESM.docx]

Search Strategy

PubMed:

(“tooth loss” OR “number of teeth” AND “hypertension”) and (“tooth loss” OR “number of teeth” AND “blood pressure”)

247

EMBASE:

(“tooth loss” OR “number of teeth” AND “hypertension”) and (“tooth loss” OR “number of teeth” AND “blood pressure”)

308

Scopus:

(“tooth loss” OR “number of teeth” AND “hypertension”) and (“tooth loss” OR “number of teeth” AND “blood pressure”)

43

Web of Science:

(“tooth loss” OR “number of teeth” AND “hypertension”) and (“tooth loss” OR “number of teeth” AND “blood pressure”)

57
